# The assessment of renal function in relation to the use of drugs in elderly in nursing homes; a cohort study

**DOI:** 10.1186/1471-2318-11-1

**Published:** 2011-01-11

**Authors:** Sara Modig, Christina Lannering, Carl Johan Östgren, Sigvard Mölstad, Patrik Midlöv

**Affiliations:** 1 Department of Clinical Sciences in Malmö, Center for Primary Health Care Research, Lund University, Malmö, Sweden; 2 Unit of Research and Development in Primary Care, Futurum, Jönköping, Sweden; 3 Department of Medical and Health Sciences, Primary Care, Linköping University, Linköping, Sweden

## Abstract

**Background:**

Renal function decreases with age. Dosage adjustment according to renal function is indicated for many drugs, in order to avoid adverse reactions of medications and/or aggravation of renal impairment. There are several ways to assess renal function in the elderly, but no way is ideal. The aim of the study was to explore renal function in elderly subjects in nursing homes and the use of pharmaceuticals that may be harmful to patients with renal impairment.

**Methods:**

243 elderly subjects living in nursing homes were included. S-creatinine and s-cystatin c were analysed. Renal function was estimated using Cockcroft-Gault formula, Modification of Diet in Renal Disease (MDRD) and cystatin C-estimated glomerular filtration rate (GFR). Concomitant medication was registered and four groups of renal risk drugs were identified: metformin, nonsteroidal anti-inflammatory drugs (NSAID), angiotensin-converting enzyme -inhibitors/angiotensin receptor blockers and digoxin. Descriptive statistics and the Kappa test for concordance were used.

**Results:**

Reduced renal function (cystatin C-estimated GFR < 60 ml/min) was seen in 53%. Normal s-creatinine was seen in 41% of those with renal impairment. Renal risk drugs were rather rarely prescribed, with exception for ACE-inhibitors. Poor concordance was seen between the GFR estimates as concluded by other studies.

**Conclusions:**

The physician has to be observant on renal function when prescribing medications to the elderly patient and not only rely on s-creatinine level. GFR has to be estimated before prescribing renal risk drugs, but using different estimates may give divergence in the results.

## Background

Drug elimination through the kidneys is normally impaired in the elderly, both due to reduced renal blood flow and perturbations in glomerular filtration rate (GFR) [1]. In addition, elderly patients have much comorbidity, such as hypertension, diabetes, and atherosclerotic disease, which contribute to reduced renal function. This is the most important pharmacokinetic alteration in the elderly. Most drugs and their active metabolites are eliminated through the kidneys. Therefore dosage adjustment according to renal function is indicated for many drugs, in order to avoid accumulation of the drugs or their metabolites, adverse reactions and/or aggravation of renal impairment [2]. However, it has been shown that these adjustments are inadequately made by clinicians [3,4].

There is no ideal way to assess renal function in the elderly. Serum creatinine level alone is often very misleading. Many geriatric patients with a "normal" serum creatinine level actually have a moderate renal impairment when GFR is estimated [4,5]. The use of GFR estimation equations, such as the Modification of Diet in Renal Disease (MDRD) or the Cockcroft and Gault formula (CG), should increase the awareness among physicians about the accuracy of renal function [6]. However, there is a variation in creatinine metabolism among these comorbid patients [7]. The use of cystatin C for estimating GFR may be a better alternative, since it is not affected by the muscle volume [8]. There are however studies showing that cystatin C is not independent of body composition [9]. S-cystatin C seems to be a useful marker for estimating GFR in the elderly [10].

There are many common pharmaceuticals that may be harmful to patients with renal impairment. Metformin is the first-line drug of choice for patients with type 2 diabetes mellitus with beneficial effects on insulin resistance and hyperglycaemia [11]. However, like all drugs it also has side effects and the main concern with metformin is the elevated risk of lactic acidosis. This risk can be minimized by avoiding the drug or adjusting the dose in patients with renal impairment, advanced coronary or lung illness, or concomitant use of contrast media.

Nonsteroidal anti-inflammatory drugs (NSAID) are widely used medications in the treatment of musculoskeletal disorders, also among elderly [12]. This may be inadequate, especially when used on regular basis, since the risk of renal toxicity is obvious in patients with reduced renal function [2,13,14]. However, even after an occasional intake of diclofenac, the renal function in healthy elderly is impaired [15]. Hence, the kidneys are not only responsible for changes in pharmacokinetics, but also the target organ of adverse reactions [2].

Angiotensin-converting enzyme inhibitors (ACEI) are used for the treatment of congestive heart failure, hypertension, for secondary prevention after myocardial infarction and for slowing the progression of renal disease [16]. Hyperkalemia does not usually occur in renocompetent patients, but can be common in patients with renal impairment, diabetes and those taking medications which interfere with renal potassium secretion. Therefore potassium levels should be monitored carefully in patients at risk. There is no specific creatinine level that is an absolute contraindication to ACEI therapy. However, it is recommended to titrate the dosage slowly when initiating treatment and the target dosage may have to be reduced [16]. An initial increase of 20 percent in the serum creatinine levels is common, but it is not an indication for discontinuing the medication. The same cautions apply when prescribing angiotensin receptor blockers (ARB) as ACEI.

Digoxin is used as symptomatic treatment in subjects with heart failure and atrial fibrillation. Morbidity associated with the use of digoxin is common due to its narrow therapeutic index. The toxicity of digoxin is dose dependent and is often the result of alterations in renal function [17]. In patients with renal impairment, digoxin therapy is associated with an increase in the risk of cardiac dysrhythmias [18]. For this reason it is recommended to reduce the dose in these patients and the serum concentration must be monitored carefully.

There is not much research on the population of frail elderly in nursing homes, although these patients often have both many medications and reduced renal function. The aim of this study was to explore the renal function in elderly subjects in nursing homes by using different GFR estimates and to investigate the association between the estimates. We also (and firstly) wanted to assess the use of pharmaceuticals that may be harmful to patients with renal impairment. Did the clinicians take notice of the impaired renal function before prescription?

## Methods

### Study population

This study was performed in three municipalities in southern Sweden: Jönköping, Linköping and Eslöv, as a part of a larger project, SHADES (Study on Health And Drugs in Elderly in nursing homes in Sweden). SHADES is a cohort study, which describes and analyzes mortality, morbidity and use of pharmaceuticals in people living in nursing homes in Sweden [19]. The aim of SHADES is to use the results to plan interventions giving better health, less adverse drug reactions and reduce the number of unplanned hospital admissions. All participants in the present study were elderly with multiple comorbidities, living in nursing homes and aged 65 years or more. All subjects living in the 11 nursing homes were invited to participate in the study with the exclusion criteria of severe illness/palliative care or language problems. The baseline comprehensive assessment was performed between March 2008 and September 2009, when 243 of the 315 subjects in the SHADES study were included

### Method

A nurse in each municipality respectively performed the investigations, which comprised data on concomitant medication, diagnoses, blood pressure and hospital stays. Many other variables were investigated for the main project. Blood samples for analyzes of s-creatinine and s-cystatin C were taken at the time of inclusion. S-creatinine concentrations were measured using the two-point, fixed-time kinetic Jaffé reaction on an ADVIA 1800 automated analyzer (Siemens, Deerfield, IL, USA) and calibrated to isotope dilution mass spectrometry (IDMS)-traceable values. S-cystatin C concentrations were measured using a fully-automated, rapid, particle-enhanced turbidimetric immunoassay on an ADVIA 1800 automated analyzer (Siemens, Deerfield, IL, USA). Reference values for s-creatinine were considered ≤90 μmol/l for women and ≤100 μmol/l for men. Patient's weight was measured. We used the modified MDRD [20] and the Cockcroft and Gault formulae (CG) for the estimation of renal function from creatinine. The CG-value was adjusted for body surface area calculated by the Mosteller's formula [21]. GFR was also estimated from cystatin C, using the Grubb formula [22]. In the analyzes we set cut-off points for GFR at 60 ml/min and 30 ml/min respectively, in order to harmonize with the National Kidney Foundation staging of chronic kidney disease (CKD): GFR > 60 ml/min stage 1+2 (normal renal function and mild reduction), GFR 59-30 ml/min stage 3 (moderate reduction) and GFR < 30 ml/min stage 4+5 (severe reduction and renal failure) [23].

Within the medication list, four medication groups were selected for analysis and correlation to renal function: metformin, NSAIDs, ACE-inhibitors/ARBs and digoxin. These groups were selected due to an established need for dosage adjustment or avoiding according to renal function and being quite commonly prescribed in elderly patients. Renal function was in this analysis estimated from cystatin C, since this estimate is currently the most commonly used by clinicians in Sweden.

### Statistical methods

All statistical analyzes were performed using SPSS statistical package 18.0 (SPSS, Inc. Chicago, IL). We calculated kappa-value to describe concordance between the different methods of estimating GFR. Since there is an ordinal rating of eGFR, we calculated pair wise weighted k (with squared weights) by the method given by Fleiss [24]. A kappa value <0.20 is considered poor, fair for 0.21-0.40, moderate for 0.41-0.60, good for 0.61-0.80 and very good for 0.81-1.00.

### Ethics

The ethical committee at Linköping University approved the project no. M 150-07.

## Results

In all 243 patients were included. Baseline characteristics of the subjects are presented in table 1. Diagnosis prevalences are chosen and reported due to the selected investigated medication groups and their indications.

**Table 1:** Baseline characteristics of the SHADES study population 2008-2009

	Men N = 63 (26%)	Women N = 180 (74%)
Mean age/years (range)	82.8 (65-98)	85.7 (65-101)
Mean body mass index (range)	25.1 (16.5-35.9)	25.3 (12.1-41.9)
Number with diabetes mellitus	15 (24%)	34 (19%)
Number with hypertension	18 (29%)	52 (29%)
Number with heart failure	9 (14%)	24 (13%)
Number with atrial fibrillation	11 (17%)	29 (16%)
Number with arthritis/arthrosis	3 (5%)	18 (10%)
Mean number of medications (range)	7.2 (1-14)	7.1 (0-14)
Mean systolic blood pressure/mm Hg (range)	127.0 (85-210)	137.6 (80-215)
Mean diastolic blood pressure/mm Hg (range)	71.5 (30-95)	73.6 (40-110)
Mean serum creatinine level/μmol/L (range)	111.7 (57-609)^a^	81.7 (44-223)
Mean cystatin C level/mg/L (range)	1.48 (0.90-4.39)	1.37 (0.71-3.11)
Mean cystatin C- eGFR/ml/min/1.73 m^2 ^(range)	54.5 (10-101)	59.9 (13-140)
Mean eGFR (Cockcroft-Gault)/ml/min/1.73 m^2 ^(range)	51.4 (7-101)	50.5 (15-115)
Mean eGFR (MDRD)/ml/min/1.73 m^2 ^(range)	67.2 (8-118)	64.9 (18-120)

^a ^including one patient with exceptionally high level. (The average was 103.7 μmol/L without him.)

Cystatin C-estimated GFR was 58.5 ml/min/1.73 m^2 ^on average. In women with reduced renal function (cystatin C-estimated GFR < 60 ml/min/1.73 m^2^) 42 out of 95 (44.2%) had a normal serum creatinine level (<90 μmol/l). Of men, 11 out of 34 (32.4%) with reduced renal function had a normal serum creatinine level (<100 μmol/l). In all 129 subjects had a cystatin C-estimated GFR of less than 60 ml/min/1.73 m^2 ^and of those 53 (41.1%) had a normal serum creatinine level. In 30 subjects with GFR of less than 30 ml/min 4 patients had normal serum creatinine levels.

Renal function and staging of all subjects using three GFR estimates (cystatin C-estimated, Cockcroft - Gault, modified MDRD) are shown in table 2. Weighted kappa was 0.42 (95% CI 0.34-0.51) for MDRD-CG, 0.46 (95% CI 0.36-0.55) for CysC-CG and 0.60 (95% CI 0.52-0.68) for MDRD-CysC.

**Table 2:** Renal function by three different estimates

	**eGFR/CRcl (ml/min/1.73 m **^**2**^**)**		
	>60	59-30	<30
Cystatin C-estimated^a^	114 (46.9%)	99 (40.8%)	30 (12.3%)
Cockcroft-Gault^a^	66 (27.2%)	148 (60.9%)	29 (11.9%)
MDRD^a^	149 (61.3%)	82 (33.8%)	12 (4.9%)

Presented as number of patients (%)

^a ^n = 243

Metformin was prescribed to four patients (three women and one man) and all these had a GFR of more than 60 ml/min if GFR was estimated from cystatin C or with the MDRD formula. One patient taking metformin had a GFR of less than 60 ml/min (58.9) according to the Cockcroft-Gault formula. The median metformin daily dose was 1.5 g (range 1.5-2.0).

Digoxin prescription for patients in different stages of renal function is shown in table 3. In all digoxin was prescribed to 19 patients (13 women and 6 men). Of those nine patients with cystatin C-estimated GFR less than 60 ml/min/1.73 m^2^, four had normal serum creatinine levels. The median digoxin daily dose was 0.13 mg (range 0.05-0.13).

**Table 3:** Number of patients in different GFR stages that were prescribed digoxin

	**eGFR/CRcl (ml/min/1.73 m **^**2**^**)**		
	>60	59-30	<30
Cystatin C-estimated^a^	10	8	1
Cockcroft-Gault^a^	6	13	0
MDRD^a^	13	6	0

^a ^n = 19

NSAID prescription for patients in different stages of renal function is shown in table 4. In all NSAID, i.e. diclofenac (median daily dose 50 mg), was prescribed for regular use to 4 patients, all women. Of those three patients with cystatin C-estimated GFR less than 60 ml/min/1.73 m^2^, one had a normal serum creatinine level.

**Table 4:** Number of patients in different GFR stages that were prescribed NSAID

	**eGFR/CLcl (ml/min/1.73 m**^**2**^**)**		
	>60	59-30	<30
Cystatin C-estimated^a^	1	2	1
Cockcroft-Gault^a^	1	2	1
MDRD^a^	2	2	0

^a ^n = 4

Prescription of ACE inhibitors/ARBs patients in different stages of renal function is shown in table 5. The most common was enalapril with a median daily dose of 10 mg (range 2.5-20). In all these medications were prescribed to 40 patients (28 women and 12 men).

**Table 5:** Number of patients in different GFR stages that were prescribed ACEI/ARB

	**eGFR/CRcl (ml/min/1.73 m**^**2**^**)**		
	>60	59-30	<30
Cystatin C-estimated^a^	14	20	6
Cockcroft-Gault^a^	11	23	6
MDRD^a^	22	14	4

^a ^n = 40

## Discussion

This study on frail elderly subjects living in nursing homes confirms that reduced renal function is common in this age group. We found that cystatin C-estimated GFR of less than 60 ml/min/1.73 m^2 ^was prevalent in more than half of the subjects. A study from Iceland indicates an even higher prevalence, showing that GFR of less than 60 ml/min occurs in more than 70% of elderly multimorbid patients [25].

In subjects with renal impairment (<60 ml/min), 46% had normal levels of s-creatinine. The clinical implication is that the assessment of renal function must not be based only on the levels of serum creatinine before prescribing drugs that may be harmful to elderly subjects with impaired renal function. In a hospital setting, Corsonello et al found that older patients frequently have impaired renal function despite normal serum creatinine levels and this concealed renal insufficiency leads to an increased risk of adverse drug reactions to hydrosoluble drugs [26]. Fehrman-Ekholm and Skeppholm showed that s-creatinine does not correlate with age and should therefore not be used as a measurement of renal function [27]. Furthermore, we found varying results of GFR when using different estimates. The concordance was moderate to good between MDRD and cystatin C and moderate concordance between the C-G and Cystatin C as for C-G and MDRD. The lack of good concordance between the estimates has also been shown previously [6,25,28]. Normal renal function, when considered as a GFR more than 60 ml/min, varied in this study from 27% of the patients when using the Cockcroft-Gault equation, to 61% when applying the MDRD formula. This is in accordance with previous findings, where the Cockcroft-Gault equation was shown to underestimate GFR in the elderly [27,29]. The National Kidney Disease Evaluation Programme does not recommend use of the MDRD equation in individuals with unstable creatinine concentrations, which includes patients with serious co-morbid conditions and hospitalized patients, particularly those with acute renal failure [23]. Consequently any creatinine-based estimating equation is unreliable when the patient is not in a steady state. This is important to note, as these patients often use many drugs and renal function has to be evaluated before starting therapy. Cystatin C-estimated GFR is probably a more accurate measure of renal function in this frail population [29]. However, cystatin C level may be elevated in patients with diabetes, thyroid disease and in those with elevated levels of inflammatory markers [30] as well as in those treated with steroids (15 patients in this study with median daily dose of 7.5 mg prednisolone) [31]. In Sweden, electronic reporting of cystatin C-estimated GFR has been implemented in some regions, but it was not available to all communities that were included. A new, possibly valuable estimation equation of GFR is CKD-EPI developed by Levey et al [32], but this is not used in clinical practise in Sweden. It is obvious that the divergences between the estimates may cause different prescribing patterns and also more or less caution with renal risk drugs or varying dosage adjustment depending on which estimate of GFR the physician uses.

In this study population, the investigated renal risk drugs were rather rarely prescribed, especially metformin (4 patients) and NSAID (4 patients). This may indicate awareness among the prescribers of renal impairment in the elderly population and the accompanied risk when prescribing medications. The awareness of this risk of metformin appears higher than previously reported. In an out-patient clinic in Palestine 60% of the patients treated with metformin had at least one contraindication [33] and a Thai study showed that over 80% of patients with type 2 diabetes with contraindication for metformin were using this medication [34].

The exception in our study is ACE-inhibitors/ARB, which were prescribed to 40 patients. These medications however, are also renoprotective and should thus be considered for this group of patients. Many of the subjects using ACE-inhibitors/ARB had reduced GFR (65% when cystatin C-estimated). This may represent an intentional use for these patients as renoprotection. However, one has to be observant and the potassium levels should be strictly monitored. The use of diuretics was frequent, 100 patients, and this may of course also affect potassium levels.

Almost half of the subjects taking digoxin had reduced GFR (<60 ml/min). This may be risky, due to the narrow therapeutic index of digoxin. There is an obvious risk that GFR is gradually decreasing even more, with the subsequent accumulation and adverse reactions. Sweileh et al found that digoxin was one of the most commonly prescribed inappropriate medications in patients with renal insufficiency [35]. Accordingly, digoxin is one of the medications frequently seen in patients admitted to hospital due to adverse reactions [36]. Probably the prescribers of the patients in our study had only observed s-creatinine before initiating the digoxin therapy, since almost half of the subjects with renal impairment had normal levels of s-creatinine. It is however not surprising that 19 patients were prescribed digoxin, since as many as 40 patients had chronic atrial fibrillation.

This study has some limitations. First, we only used different estimates for calculating GFR and did not have any true values of GFR to compare with, i.e. iohexol clearance. Second, this study investigated only the frailest and most multimorbid patients and the results cannot therefore be generalized to the entire population of elderly. On the other hand, the clinical relevance of the study is high, since these frail patients are more sensitive to adverse reactions and hence, the physician has to be extraordinary observant on renal function. It is possible that the reported diagnoses are not complete, since they are collected from the medical records and since some of the patients may be treated in relation to symptoms without a diagnosis. The included nursing homes, and hence the investigated patients, were not randomly selected but were conveniently sampled. The present study was a cross-sectional study and therefore it was not possible to state if s-creatinine was a stable value. There was however no clinical suspicion of any patient having an acute kidney injury. Since it is a cross-sectional study, we cannot know whether the NSAID use was permanent or for limited-time use. There were no details available about possible attempts to adjust the drugs for renal dosing. It is possible that drug use leading to adverse reactions was not captured, but only medications in patients who tolerate being on these renal risk medications.

Since impaired renal function is common among the elderly, further research is needed in this area. More knowledge about how to estimate renal function in a simple and precise way would be valuable. More research is also required about medications used for this population, so that guidelines for dosage, therapy initiating and possible interactions will be evidence based.

## Conclusions

We report that reduced renal function is common among frail elderly subjects living in nursing homes, although nearly half of those with renal impairment have a normal creatinine level. Lack of good concordance was found between the different GFR estimates used. In this study population, the investigated renal risk drugs were rather rarely prescribed, implicating awareness among physicians about the risks. The clinical implication of this study is that the physicians have to be cautious when prescribing medications that may be harmful to elderly subjects with renal impairment and this decision should not be based solely on serum creatinine levels. GFR has to be estimated before prescribing renal risk drugs, but using different estimates may give different results.

## Competing interests

The authors declare that they have no competing interests.

## Authors' contributions

SMO participated in the design of the study, performed the analysis and drafted the manuscript. CL, CJÖ, SMÖ and PM participated in the design of the study and revised the manuscript. All authors read and approved the final manuscript.

## Pre-publication history

The pre-publication history for this paper can be accessed here:

http://www.biomedcentral.com/1471-2318/11/1/prepub
